# Fxyd2 regulates Aδ- and C-fiber mechanosensitivity and is required for the maintenance of neuropathic pain

**DOI:** 10.1038/srep36407

**Published:** 2016-11-02

**Authors:** Stéphanie Ventéo, Sophie Laffray, Christiane Wetzel, Cyril Rivat, Frédérique Scamps, Ilana Méchaly, Luc Bauchet, Cédric Raoul, Emmanuel Bourinet, Gary R. Lewin, Patrick Carroll, Alexandre Pattyn

**Affiliations:** 1 INSERM U1051, Institut des Neurosciences de Montpellier, Hôpital St Eloi, 80 rue Augustin Fliche, 34091 Montpellier, France; 2 UMR5203, INSERM U1191, Institut de Génomique Fonctionnelle, 141 rue de la Cardonille, 34094 Montpellier, France; 3 Université de Montpellier, Place Eugène Bataillon, 34090 Montpellier, France; 4 Department of Neuroscience, Max-Delbrück Center for Molecular Medicine, Robert-Rössle Str. 10, D-13125 Berlin, Germany; 5 Département de Neurochirurgie, Hôpital Gui de Chauliac, CHU, 80 avenue Augustin Fliche, 34295 Montpellier, France

## Abstract

Identification of the molecular mechanisms governing sensory neuron subtype excitability is a key requisite for the development of treatments for somatic sensory disorders. Here, we show that the Na,K-ATPase modulator Fxyd2 is specifically required for setting the mechanosensitivity of Aδ-fiber low-threshold mechanoreceptors and sub-populations of C-fiber nociceptors, a role consistent with its restricted expression profile in the spinal somatosensory system. We also establish using the spared nerve injury model of neuropathic pain, that loss of *Fxyd2* function, either constitutively in *Fxyd2*^−*/*−^ mice or acutely in neuropathic rats, efficiently alleviates mechanical hypersensitivity induced by peripheral nerve lesions. The role of Fxyd2 in modulating Aδ- and C-fibers mechanosensitivity likely accounts for the anti-allodynic effect of *Fxyd2* knockdown. Finally, we uncover the evolutionarily conserved restricted expression pattern of FXYD2 in human dorsal root ganglia, thus identifying this molecule as a potentially promising therapeutic target for peripheral neuropathic pain management.

Molecular, behavioral and electrophysiological analyses of transgenic models have proven to be a powerful strategy for dissecting the mechanisms by which functionally diverse subtypes of first order somatosensory neurons of the dorsal root ganglia (DRG) respond to nociceptive, thermal, and mechanical stimuli. In parallel, growing knowledge of the pathological changes that occur in these neurons in disease states, including chronic pain, is driving the development of new treatments for sensory disorders^1^. Based on their etiology and the cellular and molecular mechanisms involved, chronic pain conditions are classically divided into tissue damage/inflammation-induced pain and neuropathic pain^2^. Neuropathic pain is characterized by persistent spontaneous pain and/or pain hypersensitivity including allodynia and hyperalgesia, which follows lesions or dysfunction of the somatosensory system^3^,^4^. Its origins can be multiple, partly explaining why existing treatments are only moderately effective, but generally involve pathological changes in nociceptive and innocuous touch sensing neurons leading to persistent modifications in spinal pain processing circuits^4^,^5^. Peripheral nerve damage represents a major cause of neuropathic pain which alters the electrophysiological properties of primary sensory axons, not only of injured axons but also of neighboring sensitized non-injured fibers^6^,^7^,^8^. The excitability changes that follow nerve injury may be due to alterations of the expression, distribution and/or biophysical properties of various ion channels that are essential for the maintenance of normal sensory neuron excitability^3^,^4^,^5^,^9^. Previously, we and others have reported that the expression of the *Fxyd2* gene encoding the gamma subunit of the Na,K-ATPase pump^10^,^11^ is induced postnatally and restricted in adulthood to specific neuronal subtypes of DRG neurons^12^,^13^,^14^. Recently Fxyd2 has been shown to modulate the activity of the α1-catalytic subunit of the Na,K-ATPase in non-peptidergic nociceptors and to play a role in inflammatory pain^13^. Here, by examining in detail the function of Fxyd2 in neurochemically and physiologically defined subtypes of sensory neurons on *ex vivo* skin-saphenous nerve preparations of *Fxyd2*^−*/*−^ mutant mice, we reveal that Fxyd2 is specifically required to maintain normal transduction properties of both C-fiber nociceptor populations and Aδ-fiber low-threshold mechanoreceptors (LTMRs) termed D-Hair receptors. Moreover, by assessing the implication of *Fxyd2* in the pathogenesis of neuropathic pain, we establish that this gene contributes to pain chronification. Indeed, we report that *Fxyd2*^−*/*−^ mutant mice fail to maintain mechanical allodynia over time in the spared nerve injury model of neuropathic pain. We additionally provide the proof of concept that acute injections of *Fxyd2*-siRNA in neuropathic rats efficiently alleviate pain hypersensitivity induced by peripheral nerve lesions. Finally, we uncover the evolutionarily conserved restricted expression of FXYD2 in human DRG, thus identifying this molecule as a potentially promising therapeutic target for peripheral neuropathic pain management.

## Results and Discussion

**Figure 1:**
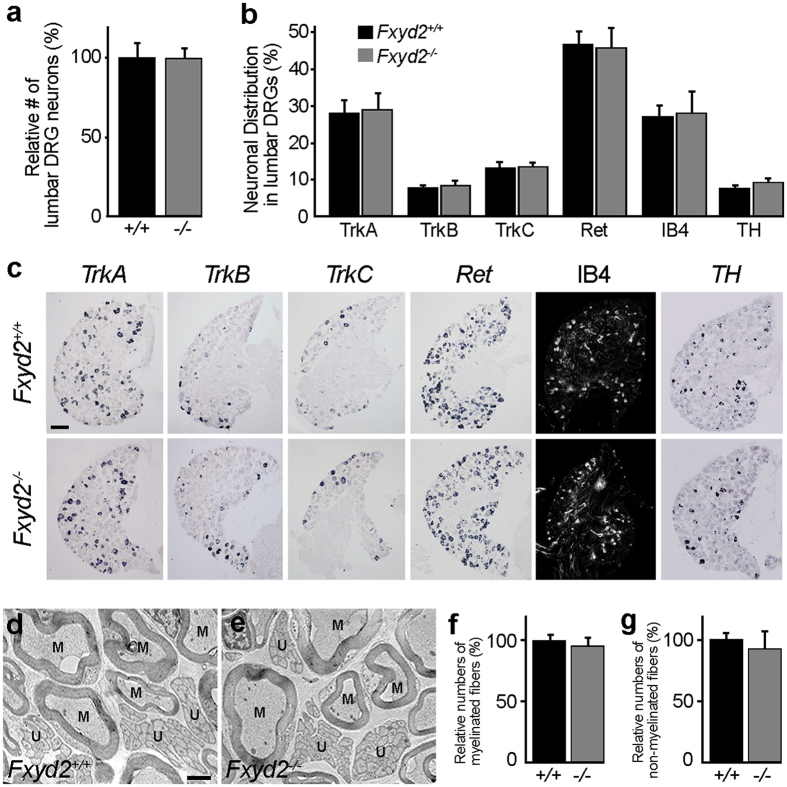
Molecular and anatomical analysis of *Fxyd2*^−*/*−^ mutant DRG and nerves. **(a)** Relative quantification of total numbers of neurons expressing the pan-neuronal marker *SCG10* in adult lumbar DRG of *Fxyd2*^+*/*+^ (+/+) and *Fxyd2*^−*/*−^ (−*/*−) animals (n = 3). (**b**) Comparative analysis of the distribution of sensory neuron subtypes expressing *TrkA*, *TrkB*, *TrkC*, *Ret*, IB4 or *TH* in *Fxyd2*^+*/*+^ and *Fxyd2*^−*/*−^ lumbar DRG (n = 3). (**c**) Representative images of *in situ* hybridization experiments using *TrkA*, *TrkB*, *TrkC*, *Ret*, or *TH* as probes and IB4 staining on cryosections of *Fxyd2*^+*/*+^ and *Fxyd2*^−*/*−^ lumbar DRG. Bar, 100 μm. **(d**,**e)** Representative electron microscopy images of the saphenous nerve from *Fxyd2*^+*/*+^ (**d**) and *Fxyd2*^−*/*−^ (**e**) mice showing myelinated (M) and unmyelinated (U) fibers. Bar, 1 μm. (**f**,**g**) Relative quantification of numbers of myelinated (**f**) and unmyelinated (**g**) fibers in the saphenous nerve of *Fxyd2*^+*/*+^ (+/+) and *Fxyd2*^−*/*−^ (−*/*−) animals (n = 3), revealing no significant difference. In (**a**,**b**,**f** and **g**), data are represented as mean ± SEM. Statistical analyses were performed using Unpaired Student’s t test.

In the spinal somatosensory system, *Fxyd2* displays a restricted expression profile in specific classes of primary sensory neurons of the DRG, including the TrkB-positive (+) D-Hair Aδ-fiber LTMRs and the IB4+ C-fiber non-peptidergic nociceptors, but is absent from second order spinal cord dorsal horn neurons (Supplementary Figure 1a–c)^12^,^13^,^14^. Analyses of *Fxyd2*^−*/*−^ animals showed that the overall number of sensory neurons was comparable to *Fxyd2*^+*/*+^ littermates (Fig. 1a), and that the proportion of the total DRG neuron subtypes that were found to be *TrkA*+, *TrkB*+, *TrkC*+, *Ret*+, IB4+ or *TH*+ was essentially unchanged in the mutants compared to controls (Fig. 1b,c). Moreover, electron microscopy analyses also established that the mean numbers of myelinated and unmyelinated fibers in the purely sensory saphenous nerve in *Fxyd2*^−*/*−^ animals were identical to those found in *Fxyd2*^+*/*+^ mice (Fig. 1d–g). Thus, *Fxyd2* appears largely dispensable for the differentiation, survival and axonal outgrowth of sensory neurons.

To assess putative physiological consequences of *Fxyd2* gene deletion, we used *ex vivo* skin-saphenous nerve preparations to analyze the properties of single sensory afferents innervating the skin in C57Bl6 WT animals (referred to as WT) and *Fxyd2*^−*/*−^ mutant mice. In these experiments, we examined the stimulus response properties of LTMRs and nociceptors with a series of quantitative stimulus protocols designed to probe the mechanosensitivity of skin afferents (see Methods and below)^15^,^16^. A summary of the receptor subtypes recorded in WT and *Fxyd2*^−*/*−^ mutants is shown in Supplementary Table 1. First, LTMRs with Aβ- or Aδ-fiber axons innervating hair follicle or Merkel cells in the skin were examined using a series of constant amplitude ramp and hold stimuli in which the speed of the ramp was systematically increased from 100 μm/s to 1000 μm/s. In WT, the firing rate of both rapidly-adapting (RAMs) or slowly-adapting (SAMs) Aβ-fiber LTMRs increased with velocity, and this was also true for RAMs and SAMs recorded from *Fxyd2*^−*/*−^ mice (Supplementary Figure 2a,c). We also measured mechanical latencies for each stimulus strength, a sensitive measure of the activation threshold^17^, and no differences in mechanical latency plots were detected between RAMs or SAMs of WT and *Fxyd2*^−*/*−^ mice (Supplementary Figure 2b,d). We also found that loss of *Fxyd2* did not significantly affect the conduction velocity of RAMs and SAMs (Supplementary Figure 2e). These data thus indicate that loss of *Fxyd2* activity does not lead to any quantitative impairment in the response properties of RAMs and SAMs, consistent with the fact that *Fxyd2* is not normally detected in these two populations.

**Figure 2:**
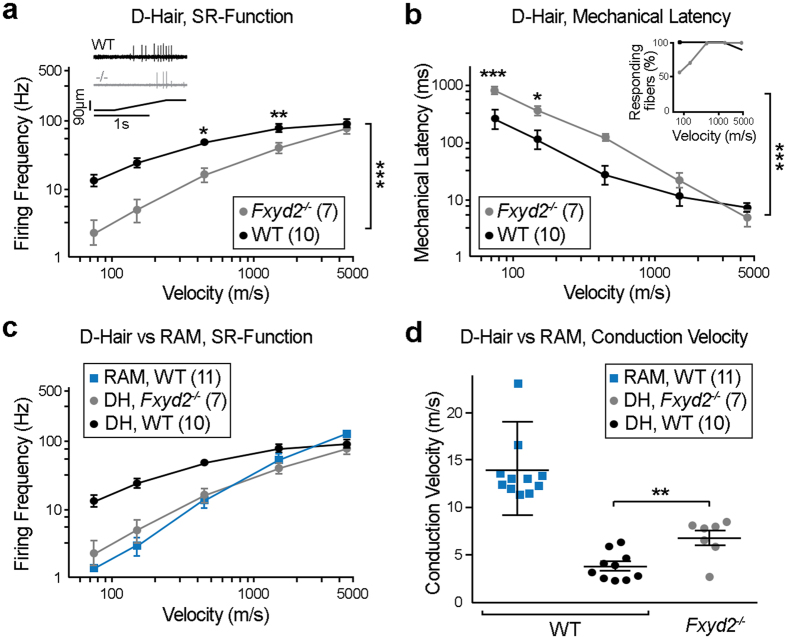
Impaired response properties of Aδ-fiber LTMRs in *Fxyd2*^−*/*−^ mutants revealed by single fiber recordings on skin-saphenous nerve preparations. **(a**,**b**,**c)** Receptive field properties of single cutaneous afferents after application of a series of ramp and hold stimuli with increasing velocities (0.075, 0.15, 0.45, 1.5 and 4.5 mm/s at 92 μm displacements) are shown for D-Hairs Aδ-fiber LTMRs (**a**–**c**) and RAMs (**c**) in WT and for D-Hairs Aδ-fiber LTMRs (**a**–**c**) in *Fxyd2*^−*/*−^ mice. Mean firing frequencies during the ramp phase of D-Hairs and RAMs (**a**,**c**) or mechanical latencies of D-Hairs (**b**) were plotted as function of stimulus velocity. Insets in (**a**) shows an illustrative examples of recordings from WT and *Fxyd2*^−*/*−^ animals, insets in (**b**) shows the proportion of fibers responding to each stimulus. (**d**) Action potential conduction velocities (CV) of D-Hairs from WT and *Fxyd2*^−*/*−^ mutants and of RAMs from WT are displayed. Note that mutant D-Hairs show untypical fast conduction properties that significantly differ from controls, though without reaching the CV of RAMs. Data are represented as mean ± SEM; numbers indicate fibers recorded. Statistical analyses were performed by Two-way ANOVA with Bonferoni post-hoc test in (**a**,**b**) and Mann-Whitney *U* test in (**d**); *P < *0.05*; **P < *0.01*; ***P < *0.001*.

In contrast, D-Hair receptors with Aδ-fiber conduction velocities, representing the most sensitive LTMR population in the skin^18^,^19^, were found to exhibit impaired stimulus-response properties in *Fxyd2*^−*/*−^ mutants. Indeed, their sensitivity to moving stimuli had a tendency to be decreased at 4 (out of 5) stimulus velocities tested in *Fxyd2*^−*/*−^ mutants compared to WT, and this was statistically significant for two of them (Fig. 2a). In the same line, mechanical latency for mutant D-Hair receptors was increased at 4 (out of 5) stimulus strengths and this was statistically significant for the first two slowest stimuli (Fig. 2b). Qualitatively, D-Hair receptors in *Fxyd2*^−*/*−^ mutant mice had stimulus-response properties that were strikingly similar to RAMs which normally have a substantially lower sensitivity, especially to slow moving stimuli (Fig. 2c). Interestingly, the mean conduction velocity of D-Hair receptors was also found to be increased in *Fxyd2*^−*/*−^ mutants (CV = 6.8 ± 0.77 m/s) compared to WT (CV = 3.8 ± 0.46 m/s) (Fig. 2d), but remained nevertheless lower than the mean conduction velocity of RAMs (13.05 ± 0.45 m/s) (Fig. 2d, Supplementary Table 1). Thus altogether, these data show that loss of *Fxyd2* function alters the electrophysiological properties of D-Hair receptors which acquire stimulus-response functions more typical, albeit not identical, to RAMs. Moreover, the fact that the proportion of Aδ-fibers found with a mechanoreceptor function was unchanged in *Fxyd2*^−*/*−^ mutant mice (Supplementary Table 1; Supplementary Figure 2f), suggests that Fxyd2 acts autonomously in this neuronal population.

We also tested the function of thinly myelinated Aδ-fiber mechanonociceptors (A-Ms) in *Fxyd2*^−*/*−^ mice but found little change in their mechanosensitivity compared to controls. There was a very slight increase in the mechanical threshold for activation of *Fxyd2*^−*/*−^ A-M fibers but this had little or no impact on their firing frequency, mechanical latency or conduction velocity (Supplementary Figure 2e,g,h).

**Figure 3:**
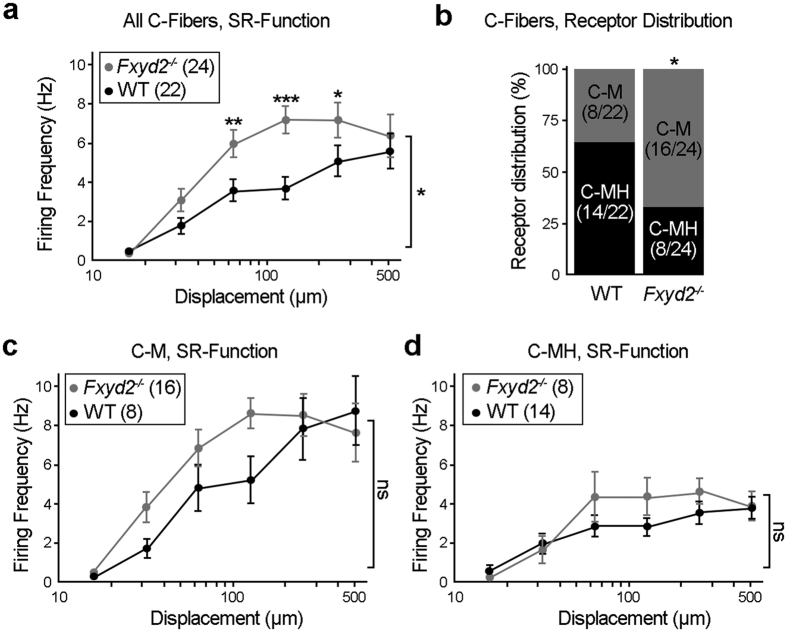
Impaired response properties of C-fiber nociceptor subtypes in *Fxyd2*^−*/*−^ mutants. **(a)** An ascending series of displacements (16–512 μm) using a constant stimulus velocity were used to mechanically stimulate C-fibers from WT and *Fxyd2*^−*/*−^ mice. Mean firing frequencies were plotted as function of displacement amplitudes. Note that globally, mutant C-fibers are significantly more sensitive to suprathreshold mechanical stimuli compared to controls. **(b)** Distribution of C-M and C-MH fibers recorded in WT and *Fxyd2*^−*/*−^ animals. The proportion of C-M vs C-MH was significantly increased in *Fxyd2*^−*/*−^ mice (36% in *Fxyd2*^+*/*+^ vs 67% in mutants). **(c,d)** An ascending series of displacements (16–512 μm) using a constant stimulus velocity were used to mechanically stimulate C-fibers identified as C-Ms (**g**) or C-MHs (h) from WT and *Fxyd2*^−*/*−^ mice. Mean firing frequencies were plotted as function of displacement amplitudes. Data are represented as mean ± SEM; numbers indicate fibers recorded. Statistical analyses were performed by Two-way ANOVA with Bonferoni post-hoc test in (**a**,**c**,**d**) and Chi-squared test in (**b**). ns: not significant; *P < *0.05*; **P < *0.01*; ***P < *0.001*.

We next assessed the physiological properties of C-fiber nociceptive neurons which encompass the Fxyd2+/IB4+ population. Interestingly, in the absence of *Fyxd2*, we found that the mechanosensitivity to suprathreshold stimuli (>50 μm) of all C-fibers recorded was significantly enhanced compared to controls (Fig. 3a). In contrast, there were no substantial differences in the mean threshold for C-fiber activation or in their response to smaller stimuli (<50 μm) between the genotypes. The majority of C-fibers, including part of the IB4+ population^20^, have been reported to be polymodal since they respond to both mechanical and noxious heat stimuli and can be classified as C-mechano-heat nociceptors (C-MHs), while the remaining mechanosensitive C-fibers not responding to noxious heat are referred to as C-mechanonociceptors (C-Ms)^21^,^22^. Strikingly, we found that the proportion of fibers classified as C-Ms or C-MHs was substantially shifted in *Fxyd2*^−*/*−^ mice. Indeed, while in control animals C-MHs and C-Ms represented, respectively, 64% and 36% of the overall C-fiber population, in *Fxyd2*^−*/*−^ mice C-Ms represented 67% and C-MHs only 33% (Fig. 3b and Supplementary Table 1). By analyzing the mutant C-Ms population which include new afferents that in WT would be normally also sensitive to noxious heat, we found that on average their firing frequencies and mechanical latency were indistinguishable from control C-Ms (Fig. 3c; Supplementary Figure 3a), and were thus clearly distinct from C-MHs (Fig. 3d; Supplementary Figure 3b). We also found that the population of fibers classified as C-MHs recorded from *Fxyd2*^−*/*−^ mice, albeit reduced in number, did not show any deficits either in their firing frequency (Fig. 3d), their mechanical latency (Supplementary Figure 3b), or in their mean firing rates to standard heat stimuli (ramp 1 °C/s, range 32–48 °C; Supplementary Figure 3c) compared to WT C-MHs. Since no neuronal loss was observed in *Fxyd2*^−*/*−^ animals (see Fig. 1a,b), these findings strongly suggest that a proportion of mutant C-MH fibers loses thermal sensitivity and becomes more sensitive to mechanical stimulation with characteristics of WT C-M fibers (Fig. 3b–d). Interestingly, C-M fibers in the saphenous nerve fire action potentials with frequencies twice as high as C-MH fibers in response to supra-threshold mechanical stimuli (compare Fig. 3c,d)^17^. Since in *Fxyd2*^−*/*−^ animals there is an increase in the proportion of nociceptors with C-M properties, this result certainly explains the enhanced sensitivity of the overall C-fiber population to mechanical stimuli observed in mutants (Fig. 3a).

These findings establishing a role for *Fxyd2* in maintaining the normal stimulus-response properties of Aδ-fiber D-Hair LTMRs and of sub-populations of C-fiber nociceptors, prompted us to test for behavioral impairments in *Fxyd2*^−*/*−^ mice, notably in response to mechanical and thermal stimuli. We first determined that *Fxyd2*^−*/*−^ mice exhibited normal locomotor coordination, as illustrated by their normal performance in the rotarod test (Supplementary Figure 4a). Next, we assessed their mechanical and thermal reflex sensitivity by using von Frey hairs and the Hargreaves test, respectively. However, in agreement with recent findings^13^, *Fxyd2*^−*/*−^ animals did not exhibit obvious impaired behavioral responses (Supplementary Figure 4b,c), thus suggesting that changes observed in single fiber recordings are either too subtle to be detected at the behavioral level or are functionally compensated.

**Figure 4:**
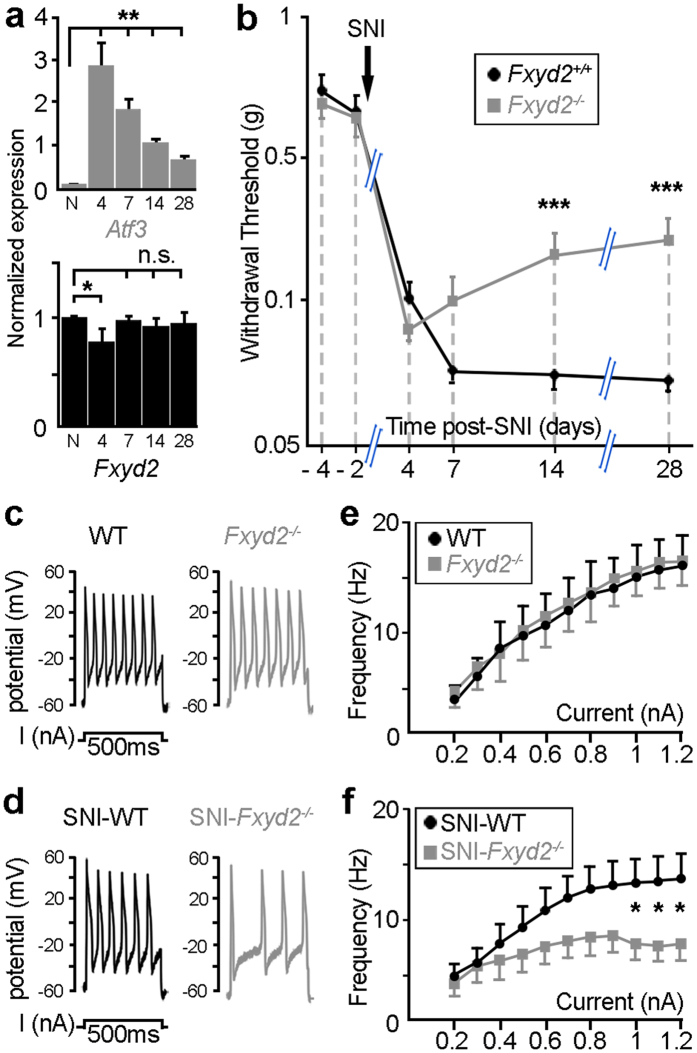
Reduced neuropathic pain in spared nerve injury-*Fxyd2*^−*/*−^ mice correlates with altered excitability of sensitized mutant IB4^+^ C-nociceptors. (**a**) RT-qPCR analysis of *Atf3* and *Fxyd2* on lumbar DRG samples from naive (N) and SNI-animals harvested between 4 and 28 days after surgery. Specific expression of *Atf3* in DRG of SNI-mice attests to their injured state. At day 4 post-SNI a slight dip in *Fxyd2* expression is observed, although levels returned to normal by day 7 and subsequently (n = 3 replicates). **(b)** Analysis of mechanical sensitivity assessed by the von Frey “up and down” method in *Fxyd2*^+*/*+^ (n = 12) and *Fxyd2*^−*/*−^ (n = 12) mice before and up to 28 days after SNI, showing selective reduction of mechanical allodynia in mutants. **(c**,**d)** Representative traces of repetitive firing elicited by 500 ms depolarizing current injection of 1 nA in IB4^+^ nociceptors from naive WT or *Fxyd2*^−*/*−^ (**c**) and SNI-WT or SNI-*Fxyd2*^−*/*−^ mice 17 days post-surgery (**d**). **(e**,**f)** Quantitative analyses of the firing frequency of IB4^+^ nociceptors from naive WT and *Fxyd2*^−*/*−^ animals (**e**) and of sensitized IB4^+^ neurons from SNI-WT and SNI-*Fxyd2*^−*/*−^ mice (**f**) after progressive increase of current intensity from 0.2 to 1.2 nA, showing selective altered excitability in SNI-*Fxyd2*^−*/*−^ animals (n = at least 10 from three distinct animals). Data are represented as mean ± SEM. Statistics were performed by one-way ANOVA and Newman-Keuls post-hoc test in (**a**) and by two-way ANOVA and Bonferroni post-hoc test in (**b,e,f**): *P < 0.05; **P < 0.01; ***P < 0.001; n.s., not significant.

We nevertheless reasoned that functional consequences of *Fxyd2* gene deletion in sensory neurons might be exacerbated in the context of a challenged somatosensory system. We thus subjected *Fxyd2*^−*/*−^ and *Fxyd2*^+*/*+^ mice to the spared nerve injury (SNI) model of peripheral neuropathic pain in which the tibial and common peroneal nerves are lesioned, while the sural nerve is left intact^23^. First, since we have reported that *Fxyd2* was durably down-regulated in sensory neurons after complete axotomy of the sciatic nerve^12^, we assessed its expression in the SNI model. In contrast to axotomy, *Fxyd2* expression was found to remain largely intact over time after SNI, though a slight and transient decreased was observed 4 days after surgery (Fig. 4a). This observation thus validated the use of the SNI model for behavioral analyses. By assessing paw withdrawal thresholds with calibrated von Frey hairs, we observed that both SNI-*Fxyd2*^+*/*+^ and SNI-*Fxyd2*^−*/*−^ mice rapidly displayed comparable mechanical allodynia. In SNI-*Fxyd2*^+*/*+^ mice, mechanical pain hypersensitivity was maintained at least 28 days following surgery. In contrast, in SNI-*Fxyd2*^−*/*−^ animals paw withdrawal thresholds started to progressively approach values found in uninjured animals beginning around day 7 post-injury (Fig. 4b). These results thus indicate that *Fxyd2* is selectively involved in the maintenance of chronic mechanical hypersensitivity induced by peripheral nerve lesions.

In order to provide insights into how *Fxyd2* loss may trigger this effect, we focused on IB4+ C-fiber nociceptors. Indeed, these neurons are Fxyd2+, are easily identifiable *in vitro* for electrophysiological recordings^20^, are functionally modified after SNI^8^ and have been previously implicated in injury-induced pain^7^,^24^,^25^,^26^. We first assessed the excitability properties of this neuronal population from uninjured WT and *Fxyd2*^−*/*−^ animals, using whole cell patch clamp recordings *in vitro*. Under control conditions, 500 ms step increases in current injection evoked a progressive increase in action potential burst frequency and no significant difference in current evoked spikes were observed between WT and *Fxyd2*^−*/*−^ neurons (Fig. 4c–e). This absence of excitability changes of mutant IB4+ cell bodies contrasted with the altered response of C-fiber subtypes following natural stimulation of their receptor fields, supporting the view that Fxyd2 mainly acts in the periphery. These data also raise the possibility that it is differences in the mechanotransduction apparatus and not electrical excitability that underlies the different receptor properties of C-M and C-MH fibers. In contrast, after SNI, the number of spikes evoked in SNI-*Fxyd2*^−*/*−^ IB4+ neurons was strikingly significantly lower than in SNI-WT neurons (Fig. 4d,f) thus illustrating the implication of Fxyd2 in the electrophysiological changes occurring after peripheral nerve lesions. These findings are reminiscent of the role of *Fxyd2* in modulating the activity of the Na,K-ATPase pump after inflammation^13^. Thus beneficial outcomes of constitutive *Fxyd2* gene deletion on neuropathic pain symptoms correlate with altered excitability of sensitized IB4^+^ non-peptidergic nociceptors. However, beyond the clear electrophysiological alterations of this neuronal population under SNI conditions, it is not totally excluded that other *Fxyd2*+ neurons might also be involved. In particular, indirect evidence has suggested that TH+ C-LTMRs^27^ may participate in the process of neuropathic pain^28^, although this issue is still controversial^29^,^30^. Also, under SNI conditions normally innocuous mechanical stimuli provoke paw withdrawal, and the absolute magnitude of the stimuli that are apparently painful in such circumstances would efficiently activate LTMRs^22^, but not most mechanosensitive C-fiber nociceptors^8^. We have shown that loss of *Fxyd2* function triggers a radical reduction in the sensitivity of D-Hair receptors that are normally the most sensitive mechanoreceptor sub-class (Fig. 2a,b)^19^. It is thus possible that allodynia might be partly also driven by activation of D-Hair receptors, but the reduction in their activation in *Fxyd2*^−*/*−^ mutants may lead to the partial alleviation of allodynia especially late in the time course of the SNI-induced hypersensitivity.

**Figure 5:**
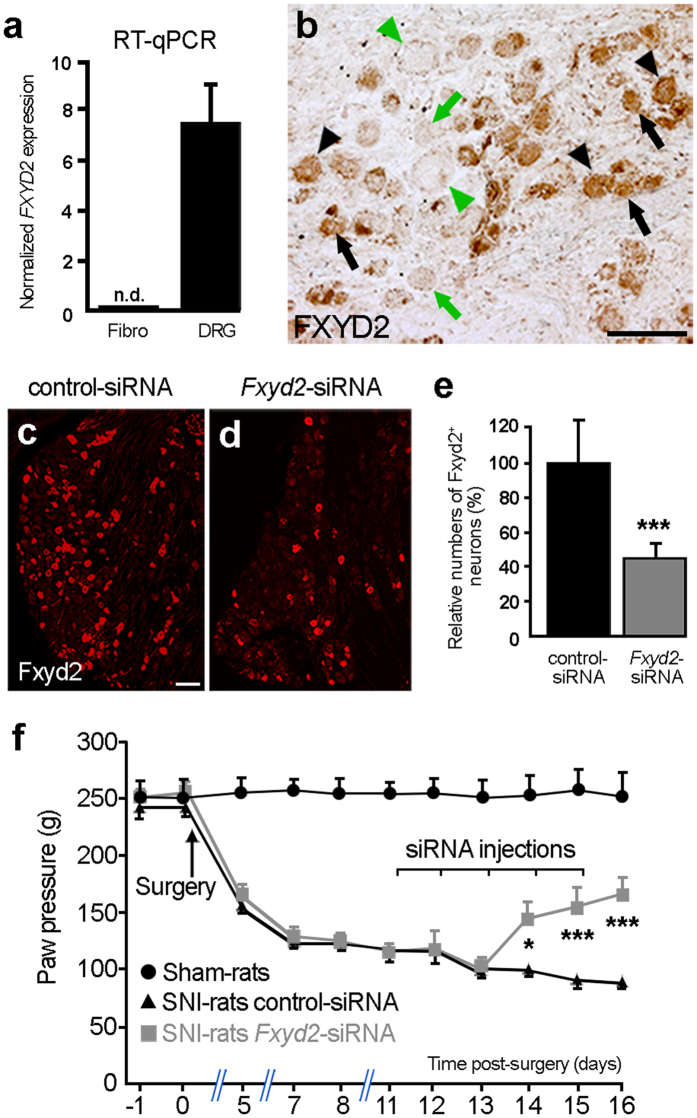
Conserved restricted expression of FXYD2 in human DRG and alleviation of mechanical pain hypersensitivity in neuropathic rats by acute intrathecal injections of *Fxyd2*-siRNA. (**a**) RT-qPCR analysis of *FXYD2* expression on human fibroblasts and human DRG samples revealing specific detection of *FXYD2* in the DRG (n = 3 replicates). (**b**) Immunohistochemistry on human lumbar DRG transverse sections using an anti-FXYD2 antibody. FXYD2 is detected in subpopulations of sensory neurons of small- (black arrows) and medium-diameters (black arrowheads). Green arrows and arrowheads point to FXYD2-negative small and large diameter neurons, respectively. (**c**,**d**) Representative images of immunofluorescent staining using an anti-Fxyd2 antibody on transverse DRG sections from SNI-rats 16 days post-surgery, that were subjected to daily injections of control-siRNA (**c**) or *Fxyd2*-siRNA (**d**) from days 11 to 15, illustrating reduced numbers of Fxyd2^+^ neurons selectively in *Fxyd2*-siRNA-injected animals. (**e**) Relative quantification of Fxyd2^+^ sensory neurons in lumbar DRG from control-siRNA-injected (n = 3) and *Fxyd2*-siRNA-injected SNI-rats (n = 3) 16 days post-surgery, that were subjected to daily injections of control- (**c**) or *Fxyd2*-siRNAs (**d**) from days 11 to 15, revealing a 60% reduction in *Fxyd2-siRNA*-injected animals. (**f**) Behavioral analyses using the Randall-Selitto test on Sham-rats (n = 6, black curve with circles) and SNI-rats injected either with control- (n = 6, black curve with triangles) or *Fxyd2*-siRNA (n = 6, grey curve with squares) after the establishment of chronic pain, showing selective pain alleviation by *Fxyd2*-siRNA injections. Data are represented ± SEM. Statistics were performed by Unpaired Student’s *t* test in (**e**) and two-way ANOVA and Bonferroni post-hoc test in (**f**); n.d., not detected; *P < 0.05; ***P < *0.001*. Bars, 100 μm.

Intriguingly, the selective requirement of *Fxyd2* for the maintenance of a chronic pain state, being of inflammatory^13^ or of neuropathic origin (this study), contrasts with the apparently normal behavior of mutant mice under healthy conditions (Supplementary Figure 4)^13^. This suggests that *Fxyd2* constitutes a key determinant whose function is mainly engaged post-injury and raises the possibility that targeted inhibition of its activity and/or expression may represent a strategy to manage neuropathic pain symptoms in a clinical setting. As a prerequisite to test this issue, we assessed whether *FXYD2* expression was conserved in human DRG. First, by RT-qPCR analyses on human cDNA samples prepared from fibroblasts or lumbar DRG, we observed that *FXYD2* was present at high levels specifically in the DRG (Fig. 5a). In addition, by performing immunohistochemistry on human lumbar DRG sections, we found that FXYD2 was strikingly detected in subpopulations of sensory neurons of small- and medium-diameters representing about 45% of the entire DRG neuronal population (Fig. 5b) consistent with what was observed in rodents. Therefore, these results show that FXYD2 expression in primary sensory neuron subtypes is conserved between mouse and man.

Next, to begin to explore the therapeutic potential of blocking *Fxyd2* function *in vivo*, we tested whether acute intrathecal injections of *Fxyd2*-siRNA into the sub-arachnoid space in SNI-rats, after the establishment of a chronic pain state, might have beneficial impacts. We took advantage of the fact that the sub-arachnoid space is contiguous with the dorsal roots such that siRNA diffuses into the DRG and penetrates into the sensory neurons^31^. Using this approach, we determined that daily *in vivo* administration of *Fxyd2*-siRNA during 5 days led to a 60% reduction in numbers of Fxyd2^+^ neurons in the DRG compared to injection of control-siRNAs (Fig. 5c–e). We thus carried out behavioral analyses on neuropathic rats by studying their responses to noxious mechanical stimulations. Using the modified Randall-Selitto test^32^ based on vocalization behavior, we found that SNI-rats exhibited persistent mechanical hyperalgesia, and that injections of control-siRNA had no effect on the pathological symptoms over time. In contrast, intrathecal *Fxyd2*-siRNA injections significantly alleviated SNI-induced mechanical pain hypersensitivity in these animals starting 3 days after the first injection and continuing subsequently (Fig. 5f). Similar results were obtained by analyzing mechanical allodynia using the von Frey test (Supplementary Figure 4d). These data thus show that acute inhibition of *Fxyd2* expression has beneficial effects on neuropathic pain symptoms, thus providing the proof of concept for its therapeutic relevance.

In conclusion, these data reveal novel roles for the Fxyd2 protein in setting the mechanosensitivity of Aδ-fiber D-Hair receptors and of a sub-population of C-fiber nociceptors. They also extend previous findings by establishing that under pathological conditions, loss of *Fxyd2* either constitutively in *Fxyd2*^−*/*−^ mutant mice or acutely by intrathecal injections of siRNA in rats, efficiently alleviates neuropathic pain induced by peripheral nerve lesions. Since loss of *Fxyd2* function appears compatible with normal physiology and since this protein is not expressed in spinal neurons, it is likely that the protective effects of blocking Fxyd2 function against peripheral lesion-induced pain reflect changes in the basal sensitivity of injury-induced excitability of IB4+ nociceptors, but also possibly of D-Hair mechanoreceptors. Finally, the fact that the expression of FXYD2 protein appears conserved in human DRG suggests that it could be an interesting therapeutic target for neuropathic pain management.

## Materials and Methods

### Animals and Surgery

All experiments were approved by the Direction Départementale des Services Vétérinaires de l’Hérault (Certificate of Animal Experimentation n° 34–376, 17 February 2009) and performed according to the guidelines of the International Association for the Study of Pain (IASP). *Fxyd2*^−*/*−^ mice^11^ were maintained on a C57Bl6 background. For molecular, histological and behavioral analyses on mice, wild type littermates (referred to as *Fxyd2*^+*/*+^) were used. Wild-type C57Bl6 animals (referred to as WT) were used as controls for single neuron recordings (obtained from Janvier) or *ex vivo* skin-nerve preparations (obtained from Charles Rivers). All animals were housed with a 12/12 dark/light cycle and ad libitum access to water and food. Five week-old male Sprague-Dawley rats (Janvier), weighing 200 to 250 g at the beginning of the experiments, were used. Unilateral Spared Nerve Injury (SNI) surgery^23^ was performed on animals which were deeply anesthetized with Ketamine-Xylasine (respectively 100 mg/kg and 10 mg/kg). Briefly, the left hindlimb was immobilized in a lateral position and slightly elevated. Skin incision was made at mid-thigh level using the femur as a landmark, and muscle layers were separated to bring out the sciatic nerve and its three branches (sural, common peroneal, and tibial nerves). Both tibial and common peroneal nerves were tightly ligated with a 6.0 silk thread (Ethicon), transected together and a 1–2 mm section of the two nerves was removed. The sural branch was carefully preserved by avoiding any nerve stretch or contact with surgical tools. The muscle layer was closed by careful apposition and skin sutured with vicryl 4.0 threads. A subcutaneous injection of NaCl 0.9% (10 ml/kg) was finally performed to prevent for dehydration. Note that rats in the sham group had their sciatic nerve exposed as in the SNI procedure, but they received no further manipulations.

### *Ex vivo* skin-nerve preparations

Skin-nerve preparations to record from single primary afferents were performed as previously described^17^. Mice were killed by CO_2_ inhalation for 2–4 min followed by cervical dislocation. The saphenous nerve and the skin of the hind limb were dissected and placed in an organ bath. The chamber was perfused with a synthetic interstitial fluid (SIF buffer: NaCl, 123 mM; KCl, 3.5 mM; MgSO_4_, 0.7 mM; NaH_2_PO_4_, 1.7 mM; CaCl_2_, 2 mM; sodium gluconate, 9.5 mM; glucose, 5.5 mM; sucrose, 7.5 mM; and HEPES, 10 mM at a pH of 7.4). The skin was placed with the corium side up in the organ bath. The nerve was placed in an adjacent chamber on a mirror to aid fiber teasing under a stereomicroscope. Fine filaments were teased from the saphenous nerve and placed on the recording electrode. Electrical isolation was achieved with mineral oil. Mechanical sensitivity of single units was tested by mechanical stimulation with a glass rod. Mechanically-evoked spikes were visualized and the template saved in an oscilloscope. Then, a sharp tungsten metal electrode was placed in the receptive field and an electrically evoked spike was elicited with suprathreshold current pulses and the electrical latency, the time from the stimulation artifact to spike, was recorded. The distance between the stimulating and recording electrode was designated as the conduction distance. For each isolated fiber the conduction velocity was calculated by dividing conduction distance over electrical latency for the spike. A computer-controlled nanomotor (Kleindiek, Reutlingen, Germany) was used to apply controlled displacement stimuli of known amplitude and velocity. The probe was a stainless steel metal rod and the diameter of the flat circular contact area was 0.8 mm containing a force transducer (Kleindiek, Reutlingen, Germany). The signal driving the movement of the linear motor and raw electrophysiological data were collected with a Powerlab 4.0 system (ADInstruments) and spikes were discriminated off-line with the spike histogram extension of the software.

### Behavioral testing on mice

Locomotor coordination was evaluated with a rotarod accelerating procedure (0–16 rpm for 3 min) and the latency to fall was measured. Thermal sensitivity was determined blindly using the Hargreaves test^33^. Briefly, a radiant heat source (a high-intensity projector lamp) was focused onto the plantar surface of the paw and paw-withdrawal latency was determined. Each paw was tested 3 times with a 10 min interval between each trial, and a maximal cut-off time of 20 s was used to prevent tissue damage. Mechanical sensitivity on mice was assessed blindly by placing animals on an elevated wire mesh grid and stimulating the hind paw with von Frey hairs by using the “up and down” paradigm^34^. The left hind paw was tested twice at 30 min intervals. The schedule of the experiment was as follows: after testing for baseline mechanical sensitivity on two occasions separated by at least 24 h, mice were subjected to SNI surgery. Note that, as this model spared the sural territory, von Frey stimuli were performed on the lateral aspect of the hind paw during the baseline and post-operative measurements. Mice were tested on postoperative days 4, 7, 14 and 28. Experiments were performed on six males and six females for each genotype. No significant difference was observed between genders.

### Intrathecal siRNA injections in rats

An “ON-TARGET plus” siRNA directed against the rat *Fxyd2* mRNA was purchased from Dharmacon. The sense sequence was as follows: 5′-AAUCCCUUCGAGUAUGAUUU-3′ 35. The siGENOME Non-targeting siRNA #2 (Dharmacon, D-001210-02-20) was used as a negative control. SNI-operated rats were daily intrathecally injected from day 11 to 15 after surgery under brief isoflurane anesthesia with 2 μg of control- or *Fxyd2*-siRNA in Exgen transfection reagent (Euromedex, ET0250) in a volume of 20 μl. The transfection mix was prepared as follows: 10 μl of siRNA solution containing 50 μg was mixed with 240 μl of 5% glucose solution in water. Exgen500 transfection reagent (9 μl) was separately added to 241 μl of glucose solution and mixed. After combining the 2 solutions, they were mixed and then left for 10 min at room temperature before injections into the sub-arachnoid space^31^.

### Behavioral testing on rats

Sprague-Dawley rat males were housed three per cage under standard conditions of light and temperature. Commercial chow pellets and tap water were available ad libitum. After arrival, animals were left to become accustomed to the colony room for 4 days. To avoid stress resulting from the experimental conditions, analyses were performed by the same experimenter in quiet conditions in a test room close to the colony room. For 2 weeks before the experiments, animals were weighed daily, handled gently for 5 min, and placed in the test room for 1 h, where they were left to become accustomed to the nociceptive apparatus. Mechanical allodynia and mechanical hyperalgesia were evaluated the day before and the day of surgery (d-1 and d0) and once daily after surgery. For mechanical allodynia, we performed the von Frey test, as described above for mice, on four animals for each experimental condition. For mechanical hyperalgesia, nociceptive thresholds in handheld rats were determined with the paw-pressure vocalization test as previously described^32^ on six animals for each experimental condition. Briefly, a constantly increasing pressure was applied to the injured hind paw until the rat squeaks. The Basile analgesimeter (Apelex; stylus tip diameter, 1 mm) was used. A 600 g cutoff value was determined to prevent tissue damage.

### Real-time qPCR

Lumbar DRG (L4–L6) from naive and SNI-mice at different days post-surgery (4, 7, 14 and 28) were dissected and stored at −80 °C until RNA was extracted using the RNAqueous-4PCR Kit (Ambion). 1 μg of total RNA from mouse DRG, from human DRG (Clontech, Lot number 1105216A) or from human fibroblasts (provided by Dr. C. Angebault-Prouteau) were reverse-transcribed with 100 U of Superscript II reverse transcriptase (Invitrogen) and 5 μM hexamer random primers (Boehringer Mannheim), 0,5 mM of each dNTPs (Pharmacia), 10 mM of dithiothreitol and 20 U of recombinant RNase inhibitor (Promega) for 1 hour at 37 °C and then stored at –80 °C until use. Real time PCR was carried out as described previously^36^ using SYBR Green I dye detection on the LightCycler system (Roche Molecular Biochemichals). PCR reactions were performed in 96 well plates in a 10 μl volume containing 3 μl of RT product (final dilution 1/30), 0.5 μM of forward and reverse primers, and 2 μl of QuantiTect SYBR Green PCR Master Mix (Roche Diagnosis). Amplified products were sequenced at least once (Beckman Coulter Genomics, UK). The relative amounts of specifically amplified cDNAs were calculated using the delta-CT method^37^,^38^ on three independent experimental replicates and normalized by dividing with an appropriate normalization factor. For mice samples, this factor was the geometric mean of two stable control genes: *polymerase (RNA) II polypeptide J (Polr2j*) and *DEAD box polypeptide 48 (Ddx48*). *Beta actin (ACTB*) was used for human samples. Sequences of the primer pairs were as follows: *Polr2j* (GenBank:NM_011293): F-ACCACACTCTGGGGAACATC, R-CTCGCTGATGAGGTCTGTGA; *Ddx48* (GenBank:NM_138669): F-GGAGTTAGCGGTGCAGATTC, R-AGCATCTTGATAGCCCGTGT; *Atf3* (Genbank: NM_007498): F-ACAACAGACCCCTGGAGATG, R-CCTTCAGCTCAGCATTCACA; mouse *Fxyd2* (Genbank: NM_007503, NM_052823): F-GGACAGAGAATCCCTTCGAG, R-CCGATTTCATTGGCAGTTG; *ACTB* (Genbank: NM_001101.3): F-GCACTCTTCCAGCCTTCCTT-; R- GTTGGCGTACAGGTCTTTGC; Human *FXYD2* (GenBank:NM_001680) primers were purchased from Clinisciences (HP205498).

### *In situ* hybridization, double *in situ* hybridization and immunohistochemistry

Simple and double *in situ* hybridization were performed as previously described^12^. Digoxigenin (DIG)- or Fluorescein-labeled antisense RNA probes were synthesized using the DIG- or Fluorescein-labelling kit (Roche), respectively. *Fxyd2, Ret*, *SCG10*, *TrkA*, *TrkB*, *TrkC*, and *TH* probes were used in this study. Immunofluorescent and isolectin-B4 staining were performed as previously described^12^. Rabbit anti-C-terminal Fxyd2 antibody (provided by Dr. S. Karlish, diluted 1:2000) and Alexa Fluor-594-conjugated secondary antibody were used (Molecular Probes, diluted 1:2000). Human lumbar DRG were collected through the “Coordination Hospitalière de Prélèvement et Agence de Biomédecine” and harvested according to the French Biomedical Agency guidelines, from two brain-dead adult patients who were organ donors, as previously reported^39^. Immunohistochemistry was performed using a mouse anti-FXYD2 antibody (Abnova, H00000486-M01) and the Vectastain ABC kit (Vector).

### Cell counting

Numbers of neurons expressing the various molecular markers of sensory neuron subtypes in *Fxyd2*^+*/*+^ and *Fxyd2*^−*/*−^ littermates, and Fxyd2+ neurons in SNI-operated rats injected either with control- or *Fxyd2*-siRNA were determined by counting cells with neuronal morphology and clearly identifiable nuclei. A minimum of six sections from lumbar DRG were counted from at least three independent animals for each genotype or conditions. The percentage of neurons expressing a given marker either over the total number of DRG neurons, or over the total number of a defined population, was calculated.

### Electron microscopy

Saphenous nerves from *Fxyd2*^+*/*+^ and *Fxyd2*^−*/*−^ littermates were dissected out and immersed in a solution of 2.5% glutaraldehyde in PHEM buffer (pH 7.4) overnight at 4 °C. They were then washed in PHEM buffer and post-fixed in a 0.5% osmic acid + 0.8% potassium ferrocyanide solution for 2 h at room temperature in the dark. After two washes with PHEM buffer, samples were dehydrated with solutions of increasing ethanol concentration (30–100%). Samples were embedded in EmBed 812 using an Automated Microwave Tissue Processor for Electronic Microscopy (Leica EM AMW). Thin sections (70 nm; Leica-Reichert Ultracut E) were collected, counterstained with uranyl acetate and lead citrate and observed using a Hitachi 7100 transmission electron microscope. Myelinated and unmyelinated fibers were counted on the saphenous nerves from three *Fxyd2*^+*/*+^ and *Fxyd2*^−*/*−^ animals.

### Mouse DRG neuron isolation and electrophysiological recordings

Naïve or SNI WT and *Fxyd2*^−*/*−^ mice were killed by CO_2_ inhalation followed by cervical dislocation and their dorsal root ganglia were harvested. Neuronal cultures were established from L4-L6 lumbar dorsal root ganglia as previously reported^40^. For identification of IB4^+^ neurons, cultures were incubated at 37 °C for 10 min with 10 μg/ml IB4-FITC (Sigma) and washed twice 5 min with the bathing solution used for electrical activity recording. After SNI, non-injured sensitized IB4^+^ neurons were identified on morphological criteria including absence of regenerative axonal growth. Action potentials in dorsal root ganglion neurons were recorded with the whole-cell patch-clamp technique after 1 day *in vitro*, at 20–22 °C. Recordings were performed on at least 10 neurons for each condition, on at least three independent animals. The bathing solution contained NaCl, 140 mM; KCl, 5 mM; CaCl_2,_ 2 mM; MgCl_2_, 1.5 mM; HEPES, 10 mM; glucose, 10 mM and the pH was adjusted to 7.4 with NaOH. Recording pipettes (3–4 MΩ) were filled with the following solution: KCl, 140 mM; HEPES, 10 mM; Mg-ATP, 2 mM; Na_2_-GTP, 0.5 mM; EGTA, 10 mM; pH 7.35, adjusted with KOH. Signals were filtered at 5 kHz and sampled at 10 kHz. Whole-cell recordings were obtained with an Axopatch 200B amplifier (Axon Instruments). The experimental parameters were controlled with a computer equipped with a Digidata 1322 analogue interface (Axon Instrument). We used pClamp software (Clampex 8.02 and 9.2; Axon Instruments) for data acquisition and analysis, respectively. Following patch membrane disruption for whole-cell recordings, only IB4^+^ neurons displaying membrane resistance above 150 MΩ were selected (300 ± 22 MΩ, n = 39). To analyze the frequency-current relationships, a 500 ms current with increasing intensities from 0.2 to 1.2 nA was injected into the neuron.

### Statistical analyses

For cell counting, statistical analyses were performed using Unpaired Student’s t test. For RT-qPCR experiments, one-way analysis of variance (ANOVA) was used followed by a post-hoc Newman-Keuls test. For behavioral studies, group and time effects were validated by two-way ANOVAs for repeated measurements. When ANOVAs showed a significant effect, Bonferroni post-hoc test was used to determine the significance of the differences. For electrophysiological analyses, data were analyzed using a two-way ANOVA, followed by Bonferroni test. Fiber conduction velocities were analyzed using the Mann-Whitney *U* test. Fiber type distribution was analyzed using the Chi-squared test. *P* values < 0.05 were considered as statistically significant. All data presented are means ± SEM.

## Additional Information

**How to cite this article**: Ventéo, S. *et al.* Fxyd2 regulates Aδ- and C-fiber mechanosensitivity and is required for the maintenance of neuropathic pain. *Sci. Rep.* **6**, 36407; doi: 10.1038/srep36407 (2016).

**Publisher’s note:** Springer Nature remains neutral with regard to jurisdictional claims in published maps and institutional affiliations.

## Supplementary Material

Supplementary Information
