# Supplementary material for: Fxyd2 regulates Aδ- and C-fiber mechanosensitivity and is required for the maintenance of neuropathic pain
Source: Sci Rep. 2016 Nov 2;6:36407. doi: 10.1038/srep36407 (PMC5090990; doi:10.1038/srep36407)
Supplement: Supplementary Information [file srep36407-s1.pdf]

Supplementary Information

Fxyd2 regulates Aδ- and C-fiber mechanosensitivity and is required for the maintenance of neuropathic pain

Stéphanie Ventéo[1], Sophie Laffray[2,3], Christiane Wetzel[4], Cyril Rivat[1,3], Frédérique Scamps[1], Ilana Méchaly[1,3], Luc Bauchet[1,5], Cédric Raoul[1], Emmanuel Bourinet[2], Gary R. Lewin[4], Patrick Carroll[1,†] and Alexandre Pattyn[1, †,*]

Supplementary Table

Mechanoreceptors			WT	*Fxyd2*$^{-/-}$
**Aβ-fibers**	RAM	Percentage Total	50% (11/22)	56% (15/27)
		CV [m/s]	13.97±1.3	13.05± 0.5
	SAM	Percentage Total	50% (11/22)	44% (12/27)
		CV [m/s]	15.2±1.2	14.2± 1.2
**Aδ-fibers**	D-hair	Percentage Total	33% (10/30)	30% (7/23)
		CV [m/s]	3.8±0.46	6.8±0.77
	AM	Percentage Total	67% (20/30)	70% (16/23)
		CV [m/s]	6.0± 0.58	5.23± 0.6
**C-fibers**	C-M	Percentage Total	37% (8/22)	67% (16/24)
		CV [m/s]	0.54± 0.07	0.59± 0.04
	C-MH	Percentage Total	63% (14/22)	33% (8/24)
		CV [m/s]	0.35± 0.03	0.5± 0.04

Supplementary Table 1. Numbers and conduction velocity of the fiber types recorded in *ex vivo* skin-saphenous preparations from WT and *Fxyd2*$^{-/-}$ animals.

Supplementary Figures and Legends

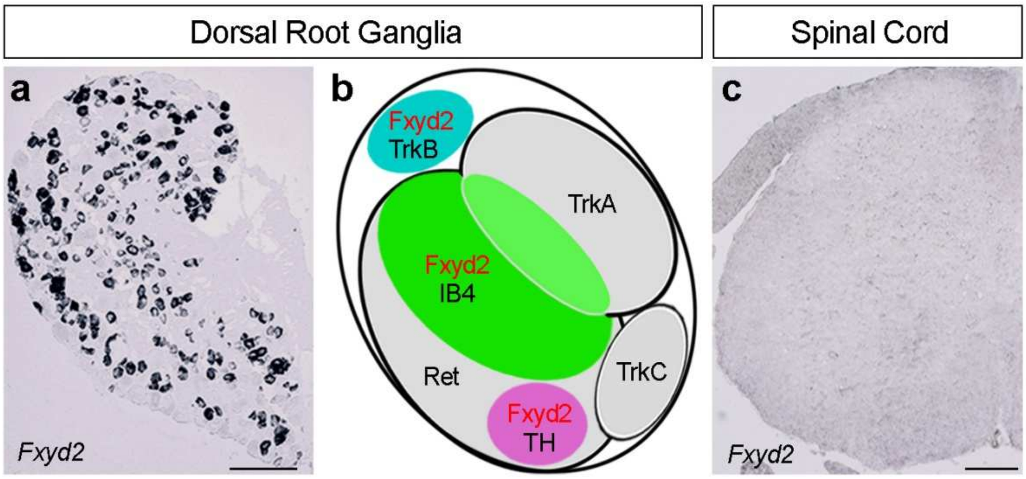

Supplementary Figure 1. *Fxyd2* expression in the spinal somatosensory system.

(a) *In situ* hybridization for *Fxyd2* on lumbar DRG sections, showing expression of *Fxyd2* in sensory neuron subtypes, representing about half of the entire neuronal population.

(b) Schematic illustration of *Fxyd2* expression in the DRG. *Fxyd2* is selectively detected in three main subtypes: the $TrkB^+$ Aδ-fiber and Ret^+/TH^+ C-fiber LTMRs, and $Ret^+/IB4^+$ C-fiber nociceptors[12-14].

(c) *In situ* hybridization for *Fxyd2* on transverse adult spinal cord hemi-section, revealing absence of *Fxyd2* expression.

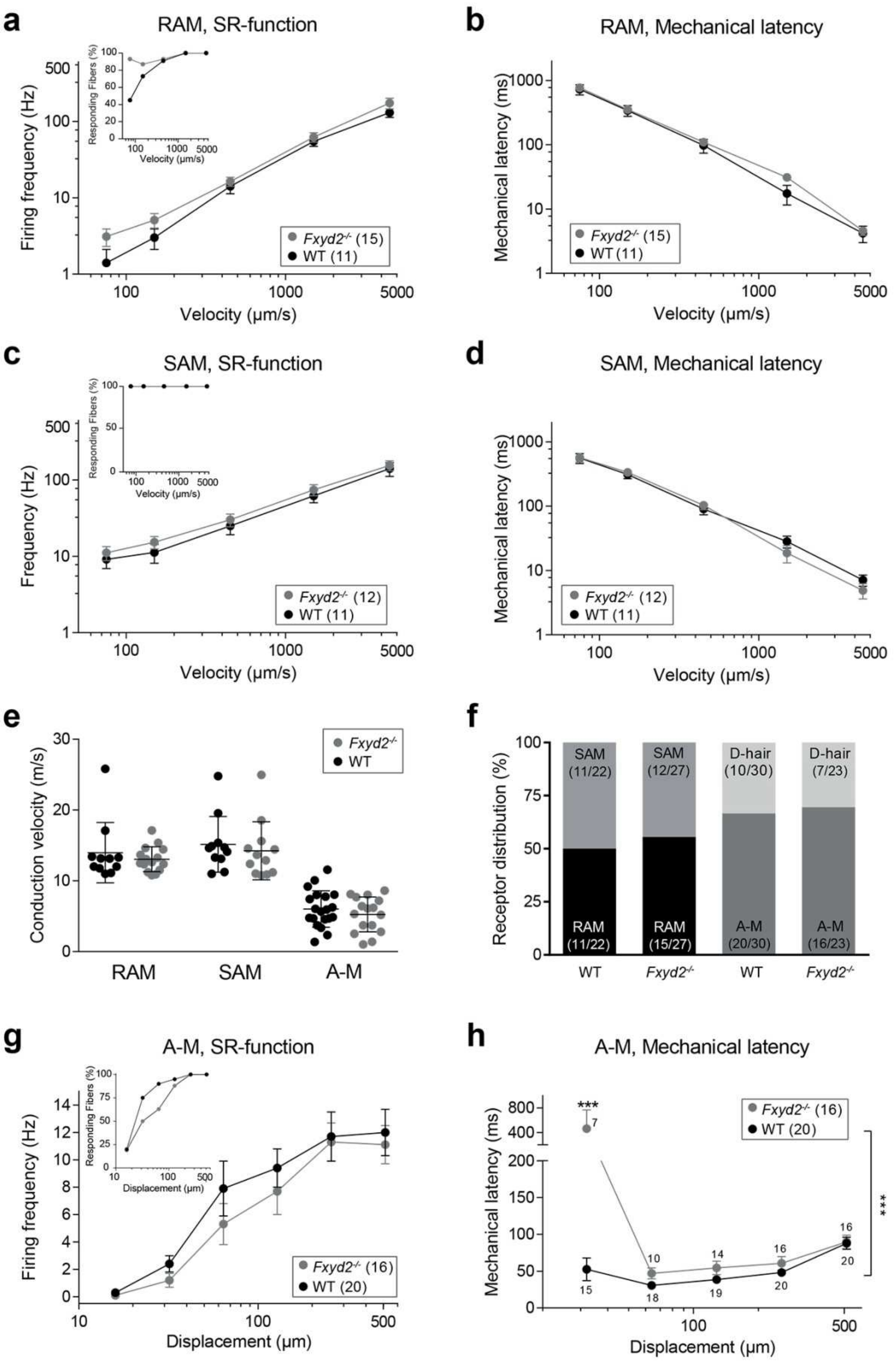

Supplementary Figure 2. Receptive field properties of single cutaneous afferents in WT and *Fxyd2*$^{-/-}$ mice.

(a-d) A series of ramp and hold stimuli with increasing velocities (0.075, 0.15, 0.45, 1.5 and 4.5 mm/s at 92 µm displacements) was applied to slowly-adapting (SAM) or rapidly adapting (RAM) mechanoreceptors from WT and *Fxyd2*$^{-/-}$ mice. Mean firing frequencies during the ramp phase (a,c) or mechanical latencies (b,d) were plotted as function of stimulus velocity. Insets in (a) and (c) show the proportion of fibers responding to each stimulus.

(e) Conduction velocities of RAM, SAM or AM fibers in WT and *Fxyd2*$^{-/-}$ mice.

(f) Distribution of RAM versus SAM fibers or D-hair versus A-M fibers recorded in WT and *Fxyd2*$^{-/-}$ animals.

(g,h) An ascending series of displacements (32–1024 µm) using a constant stimulus velocity was used to mechanically stimulate A-mechanonociceptors from WT and *Fxyd2*$^{-/-}$ mice. Mean firing frequencies (g) or mechanical latencies (h) were plotted as function of displacement amplitudes. Inset in (g) shows the proportion of AM fibers that respond to each stimulus strength.

Data are represented as mean ±SEM; numbers indicate fibers recorded. Statistical analyses were performed by Two-way ANOVA with Bonferoni post-hoc test in (a-d,g,h), Mann-Whitney *U* test in (e), Chi-squared test in (f). ***, $P<0.001$.

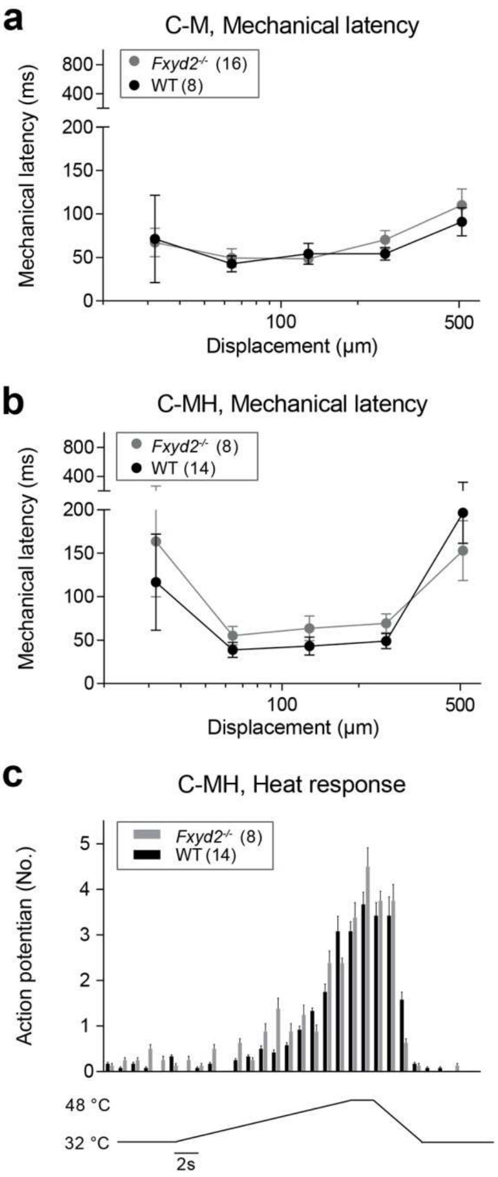

Supplementary Figure 3. Receptive field properties of C-fiber afferents in WT and *Fxyd2*$^{-/-}$ mice.

(a, b) Ascending series of displacements (32 – 1024 µm) using a constant stimulus velocity was used to mechanically stimulate C-M (a) or C-MH (b) fibers. Mechanical latencies were plotted as function of stimulus displacement.

(c) Discharge rates to standard noxious heat ramps applied to C-MH fibers recorded from WT and *Fxyd2*$^{-/-}$ mice.

Data are represented as mean ±SEM; numbers indicate fibers recorded. Statistical analyses were performed by Two-way ANOVA with Bonferoni post-hoc test.

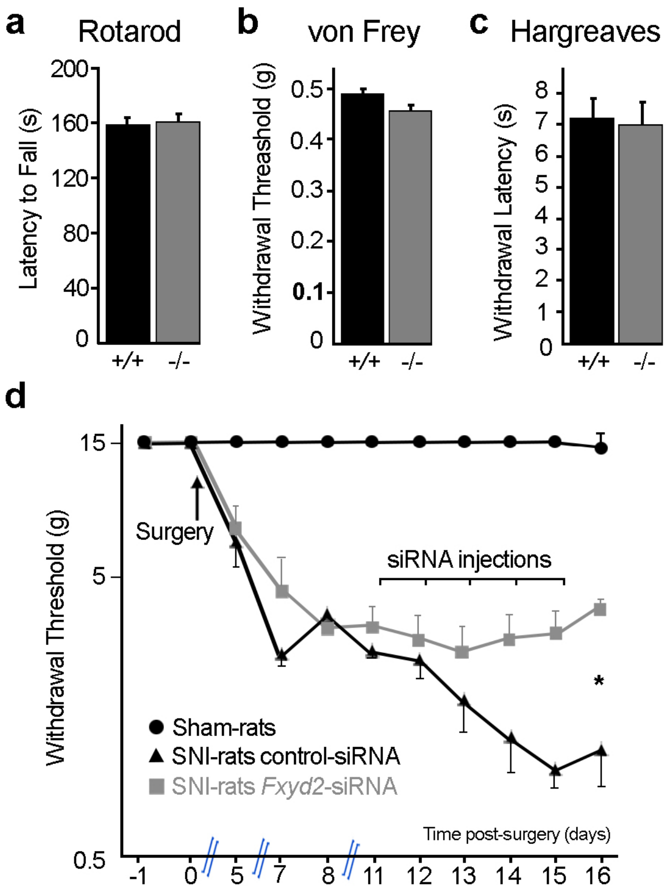

Supplementary Figure 4. Behavioral testing of *Fxyd2*$^{-/-}$ mice in healthy conditions and von Frey test on *Fxyd2*-siRNA injected SNI-rats.

(a-c) Behavioral studies in healthy conditions of *Fxyd2*$^{+/+}$ (n=12; black graphs) and *Fxyd2*$^{-/-}$ (n=12; grey graphs) mice using the locomotor rotarod test (a), the mechanical von Frey test (b) and the thermal Hargreaves test (c), revealing no significant difference between groups and genders.

(d) Behavioral analyses using the von Frey test on Sham-rats (n=4, black curve with circles) and SNI-rats injected either with control- (n=4, black curve with triangles) or *Fxyd2*-siRNA (n=4, grey curve with squares) after the establishment of chronic pain, showing selective pain alleviation by *Fxyd2*-siRNA injections.

Data are represented ±SEM. In (a-c), statistical analyses were performed using Unpaired Student's t test. In (d), statistics were performed by two-way ANOVA and Bonferroni post-hoc test; *, *P<0.05*.
